# Magnetic Nanoparticles and Drug Delivery Systems for Anti-Cancer Applications: A Review

**DOI:** 10.3390/nano15040285

**Published:** 2025-02-13

**Authors:** Willem Graham, McKayla Torbett-Dougherty, Akm Islam, Shokoufeh Soleimani, Tracy Ann Bruce-Tagoe, Jacqueline Ann Johnson

**Affiliations:** Department of Mechanical, Aerospace, and Biomedical Engineering, University of Tennessee, Knoxville, TN 37996, USA; szf775@vols.utk.edu (W.G.); mtorbet1@vols.utk.edu (M.T.-D.); aislam12@vols.utk.edu (A.I.); rfs655@vols.utk.edu (S.S.); pvd756@vols.utk.edu (T.A.B.-T.)

**Keywords:** magnetic nanoparticles, drug delivery systems, nanocarriers, drug delivery, magnetic hyperthermia, magnetic drug release, active targeting

## Abstract

Cancer continues to be a prominent fatal health issue worldwide, driving the urgent need for more effective treatment strategies. The pressing demand has sparked significant interest in the development of advanced drug delivery systems for chemotherapeutics. The advent of nanotechnology offers a groundbreaking approach, presenting a promising pathway to revolutionize cancer treatment and improve patient outcomes. Nanomedicine-based drug delivery systems have demonstrated the capability of improving the pharmacokinetic properties and accumulation of chemotherapeutic agents in cancer sites while minimizing the adverse side effects. Despite these advantages, most NDDSs exhibit only limited improvement in cancer treatment during clinical trials. The recent development of magnetic nanoparticles (MNPs) for biomedical applications has revealed a potential opportunity to further enhance the performance of NDDSs. The magnetic properties of MNPs can be utilized to increase the targeting capabilities of NDDSs, improve the controlled release of chemotherapeutic agents, and weaken the chemoresistance of tumors with magnetic hyperthermia. In this review, we will explore recent advancements in research for NDDSs for oncology applications, how MNPs and their properties can augment the capabilities of NDDSs when complexed with them and emphasize the challenges and safety concerns of incorporating these systems into cancer treatment.

## 1. Introduction

**Figure 1:**
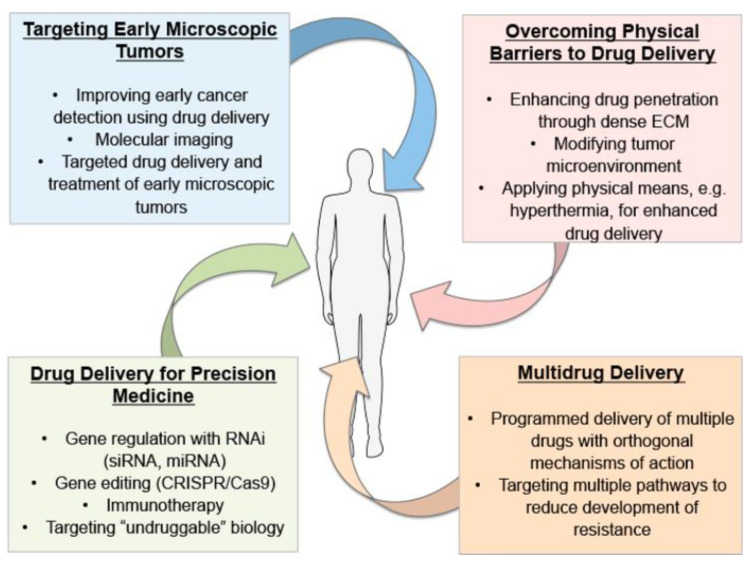
Areas of interest for drug delivery system research for oncological treatment. Adapted image obtained with permission from [5]. Copyright 2018 American Chemical Society.

According to an article by the American Cancer Society, it is projected that there will be over 2 million new cancer cases in the United States in 2024 [1]. Cancer continues to be a major health issue despite the advances in treatment, as it is the second leading cause of death in the United States alone for individuals under 85 years old [1]. This can be attributed to the ineffectiveness of current oncological therapies like chemotherapy, as these conventional treatments are frequently limited by the ability of tumors to develop drug resistance through various mechanisms [2,3]. Moreover, the cytotoxicity of chemotherapeutics often damages healthy cells and causes unwanted side effects in patients [4]. These obstacles have set a precedent for the development of more effective therapies, and the emergence of nanotechnology and nano drug delivery systems (NDDSs) could provide a promising solution for oncological treatment [2]. To improve the treatment of cancer, one avenue of research is to increase the concentration of chemotherapeutics in tumors—and drug delivery systems can accomplish this through several approaches, as shown in Figure 1.

**Figure 2:**
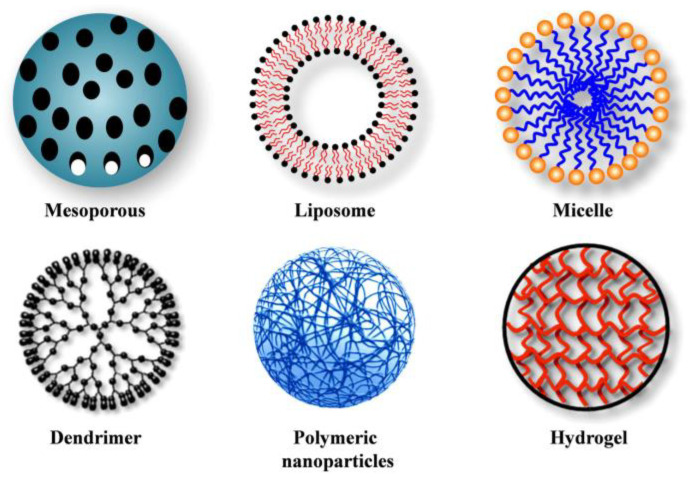
Different NDDS nanocarrier forms for oncology. Adapted image from S. Senapati et al. [12] following the Creative Commons License, (http://creativecommons.org/licenses/by/4.0/).

The development of nanomedicine-based drug delivery over the past few decades has produced several different types of NDDSs in the form of nanocarriers (NCs). These NCs (examples shown in Figure 2) are essentially nanoscale vehicles designed to enhance the facilitation of chemotherapeutic agents to cancer sites and target cells [6]. There are different forms that NCs can take, some examples include: liposomes, micelles, hydrogels, and dendrimers. Each NC is developed for certain applications in oncology, but all share the same goal of improving the efficacy of chemotherapeutics [6]. NCs can enhance the pharmacokinetics of therapeutic agents by prolonging their circulation time in the body to increase drug accumulation in cancer sites and circumvent cancer cell drug resistance [7,8]. Furthermore, while NCs can utilize “passive” targeting to proliferate in cancer cells via the enhanced permeability and retention (EPR) effect; they can also be modified to actively target cancer cells [9,10,11]. Active or selective targeting can further improve drug specificity to reduce cytotoxicity for non-cancerous cells [5]. Despite the advantages of incorporating NDDSs in oncology, there are limitations. These limitations include NC instability and premature drug release [9,12], difficulty in penetrating late-stage heterogeneous solid tumors [5], and EPR demonstrating inconsistent effectiveness in heterogeneous tumors for passive targeting [9,13]. Research is ongoing to overcome these challenges for NDDSs in cancer treatment, and a promising development is the incorporation of magnetic nanoparticles.

**Figure 3:**
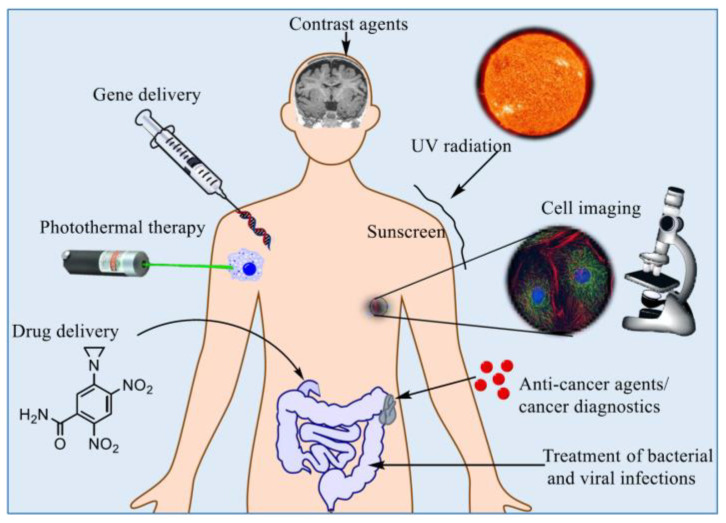
Example applications for MNPs in biomedicine. Unedited image from S. D. Anderson, V. V. Gwenin, and C. D. Gwenin [15] following the Creative Commons License, (http://creativecommons.org/licenses/by/4.0/).

While NDDSs were being researched for cancer treatment, MNPs have also been explored for applications in biomedicine. MNPs have attracted the attention of scientists and engineers for research into many fields like materials science, biotechnology, and biomedicine due to the unique physiochemical attributes they possess (example applications shown in Figure 3 [14,15]. Their controllable nanoscale size, magnetic properties, and ability to become biocompatible through coating have motivated researchers to investigate them for biomedical applications such as drug delivery since the 1970s [16]. It is important to consider that there are a variety of MNPs being researched for biomedicine and oncology, and the techniques utilized to synthesize them have a direct impact on the physical and magnetic properties of the nanoparticles [14]. It is these properties that determine the effectiveness of the MNPs in drug delivery applications. These unique properties of MNPs have inspired researchers to incorporate them into various NCs, providing several enhancements to the delivery systems. MNP integration enables features such as magnetic hyperthermia, controlled drug release by external magnetic stimuli, and imaging capabilities so the pharmacokinetics of the NDDS can be monitored [12]. The enhancements offered by MNP-coupled NCs could potentially overcome the limitations that are associated with various NDDSs, and this article will elucidate recent studies conducted that investigated NCs incorporated with MNPs for oncological drug delivery.

## 2. Magnetic Nanoparticles

**Figure 4:**
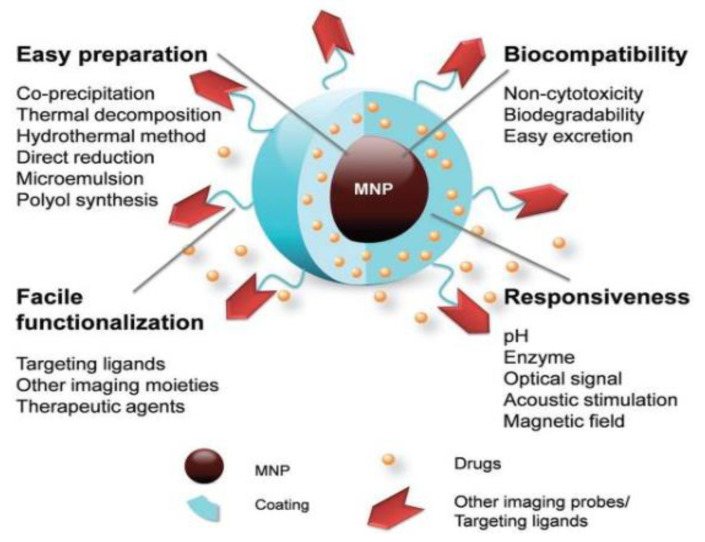
The key characteristics of MNPs position them as highly promising candidates for a wide range of biomedical applications. Unedited image from D. J. Huang et al. [20] following the Creative Commons License, (http://creativecommons.org/licenses/by/4.0/).

Magnetic nanoparticles (MNPs) are a unique class of nanoparticles (often made from materials like iron, nickel, or cobalt) and have a size between 1 and 100 nanometers [17,18]. MNPs have gained significant research attention for their promising properties across different fields such as catalysis, biomedicine, and targeted tissue therapies. In biomedical applications, MNPs are especially valuable for their small size, magnetic responsiveness, and versatility. They play critical roles in both analytical and therapeutic applications: analytically, they serve as contrast enhancers in magnetic resonance imaging (MRI). Since the subject matter of this article. Therapeutically, MNPs are utilized to deliver drugs and bioactive compounds directly to cells and to induce localized heating (hyperthermia) in cancer therapy, thereby targeting cancer cells more effectively. These versatile functions make MNPs a powerful tool in advancing biomedical research and applications [19,20]. Figure 4 depicts some of the notable features of MNPs which will be discussed later in the following sections.

**Figure 5:**
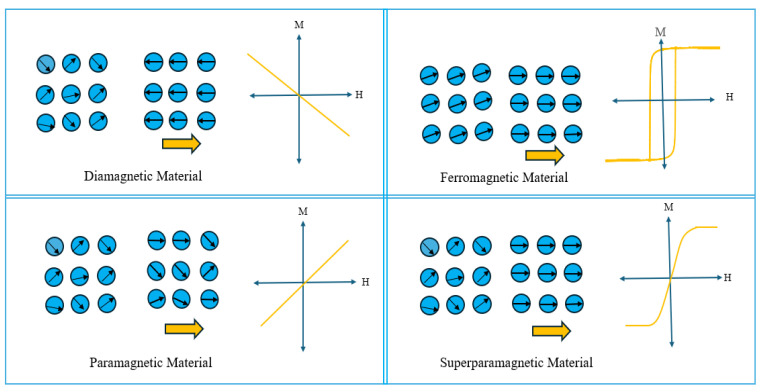
Ferromagnetic materials exhibit net magnetization without an applied field, while diamagnetic and paramagnetic materials have randomly oriented moments. Under a magnetic field, diamagnetic materials magnetize opposite to the field, whereas paramagnetic materials magnetize along its direction, both showing weak magnetism.

One of the most unique properties of magnetic nanoparticles (MNPs) is superparamagnetism [21]. This unique property is best explained by domain theory. Magnetism within any material is largely dependent on the internal atomic organization. Generally, without the presence of an applied field, the magnetization of any magnetic materials is found to be negligible. This is because magnetic materials are subdivided into some small sections known as domains which cannot be seen in the naked eye. Each of these domains have their own magnetic moments aligned in random directions without any magnetic field applied. Hence, the net magnetization is found to be very weak [22,23]. However, under the presence of an applied field, all the magnetic moments align along a specific direction and magnetic material shows strong magnetization. The alignment of the magnetic moments is applicable in a similar manner to MNPs. Nevertheless, the only thing that distinguishes the MNPs compared to bulk magnetic materials is the formation of domain walls. Essentially, the particle size of MNP is smaller than the size of the domain walls. As a result, it cannot construct magnetic domains. This feature of MNPs promotes spontaneous magnetization within the particle with respect to the applied field and makes MNPs highly controllable. Based on the configuration of magnetic moments, magnetic materials are primarily categorized as ferromagnetic, paramagnetic, and diamagnetic materials. Superparamagnetism shown by MNPs is also a form of paramagnetism. However, the magnetization in MNPs is stronger and much more spontaneous compared to the paramagnets [24]. Figure 5 shows a comparison of moment configurations and magnetization in different magnetic materials.

**Figure 6:**
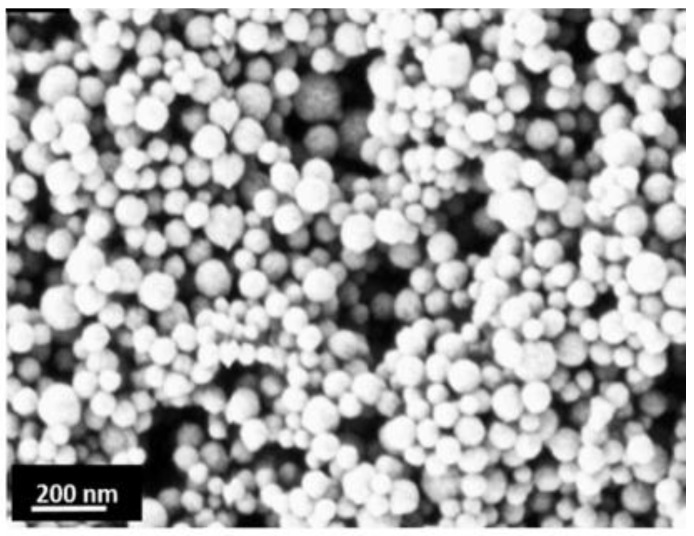
SEM image of MNPs. The image illustrates the extensive coverage achieved by the MNPs and highlights the numerous potential reaction sites they offer. Reprinted from [29], with permission from Elsevier.

Apart from their superparamagnetism, MNPs possess a high surface area-to-volume ratio that enables more extensive coating and functionalization. With a larger surface area relative to volume, MNPs have more reactive sites, facilitating rapid interactions with any therapeutic agents. With such scope of surface functionalization along with its controllability, MNPs can be employed for precise drug release to specific tissues or organs, reducing off-target effects and enhancing treatment efficiency [25]. Moreover, the controlled targeting minimizes the required dosage and decreases side effects, particularly in treatments like cancer, where localized therapy is crucial [26]. Due to these advantageous properties, magnetic nanoparticles (MNPs) have emerged as a promising tool in drug delivery applications [27]. Figure 6 shows an SEM image of MNPs that possess a large surface area to volume ratio with particle size close to 20 nm, which is smaller than the critical size of domain wall formation [28,29].

**Figure 7:**
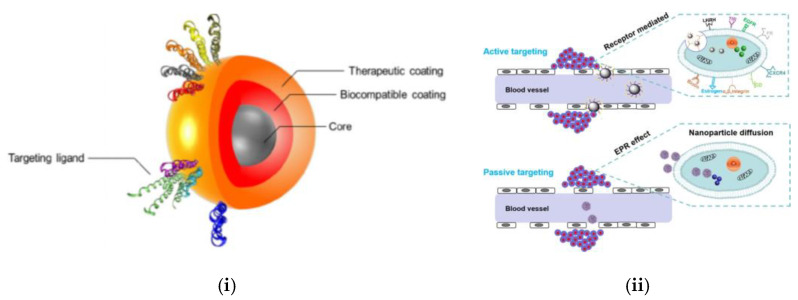
Basis of MNP hyperthermia: (**i**) Applying a biocompatible coating to ensure suitability within biological environments, and (**ii**) Employing active or passive targeting strategies tailored to the tumor’s specific characteristics or biological mechanisms. Unedited images (**i**) from H. Tian et al. [31] and (**ii**) from O. L. Gobbo et al. [34] following the Creative Commons License, (http://creativecommons.org/licenses/by/4.0/).

A major application of this controlled release is magnetic hyperthermia, an innovative cancer therapy that utilizes magnetic nanoparticles (MNPs) to produce localized heat, selectively targeting and killing cancer cells or triggering drug release [30,31]. In ferromagnets and antiferromagnets, energy applied through an external magnetic field overcomes domain wall barriers caused by intrinsic anisotropy and microstructural imperfections, creating hysteresis. Applying an oscillating magnetic field maintains a steady energy input, converted into heat, forming the basis of magnetic hyperthermia. Even though the generation of heat is much greater in ferromagnetic materials, they are not as controllable as MNPs since superparamagnetic particles can rapidly align with an external magnetic field and less prone to clumping (the tendency of MNPs to stick together due to their magnetic attraction) [32,33]. Moreover, the versatility of the functionalization process of MNPs makes it even more feasible for controlled drug delivery applications. For magnetic hyperthermia-based applications, MNPs are typically functionalized with specific molecules to target cancer cells. These functionalized nanoparticles are injected into the bloodstream, where they accumulate in tumor tissues through passive targeting (enhanced permeability and retention effect that refers to tissues’ ability to withhold nanoparticles longer than normal tissues) or active targeting (attachment to cancer-specific markers). The functionalization process is dependent on the level of specificity required to deal with the cancerous tumors [20]. Figure 7 shows the functionalization process of magnetic hyperthermia.

**Figure 8:**
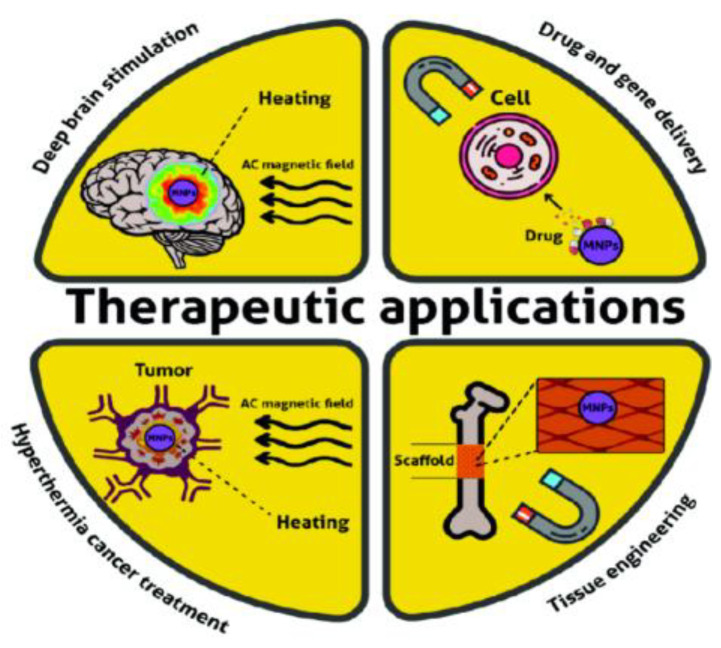
MNP based therapeutic applications: Controlled drug delivery and magnetic hyperthermia for tumor destruction are widely utilized methods, while deep brain stimulation and tissue engineering remain under active research. Unedited image from K. D. Petrov and A. S. Chubarov [41] following the Creative Commons License, (http://creativecommons.org/licenses/by/4.0/).

Magnetic nanoparticles (MNPs) show promise in tissue engineering and neuro-logical stimulation due to their controllability and surface functionalization, allowing precise control over cell positioning and patterning on bio-scaffolds for complex tissue construction [35,36]. For example, bone matrices have a highly ordered biochemical structure that MNP-laden scaffolds can help mimic. By applying magnetic fields to scaffolds infused with MNPs near the target site, MNP-treated cells can be guided to stimulate essential cellular processes such as differentiation and proliferation. This capability not only enhances the formation of intricate 3D structures but also improves scaffold stability and mechanical strength, critical in regenerating bone and cartilage tissues [37,38]. For example, osteogenic differentiation in stem cells can be accelerated with magnetic stimulation, supporting faster and more efficient bone regeneration [39]. In addition, MNP-enabled hyperthermia has demonstrated significant benefits in wound healing and cartilage repair, where localized heating activates cellular repair mechanisms. Researchers have extended this concept further by exploring MNP-driven deep brain stimulation (DBS) for neurological applications, using hyperthermia to facilitate motor function restoration. This area of research has immense potential, particularly for aiding disabled patients with impaired motor function. One notable study by Rahul et al. demonstrated that Co-Mn core–shell MNPs, introduced into the motor cortex of mice, could effectively facilitate motor activities [40]. Their results showed that magnetothermal genetic stimulation in the motor cortex evoked walking movements, while deep brain stimulation in the striatum led to rotations around the body axis. Additionally, stimulation near the boundary between the ventral and dorsal striatum triggered a freezing-of-gait response [40]. Though still in the research phase with mouse models, this application shows promise for aiding individuals with motor disabilities. Figure 8 summarizes MNP-based therapeutic applications.

**Figure 9:**
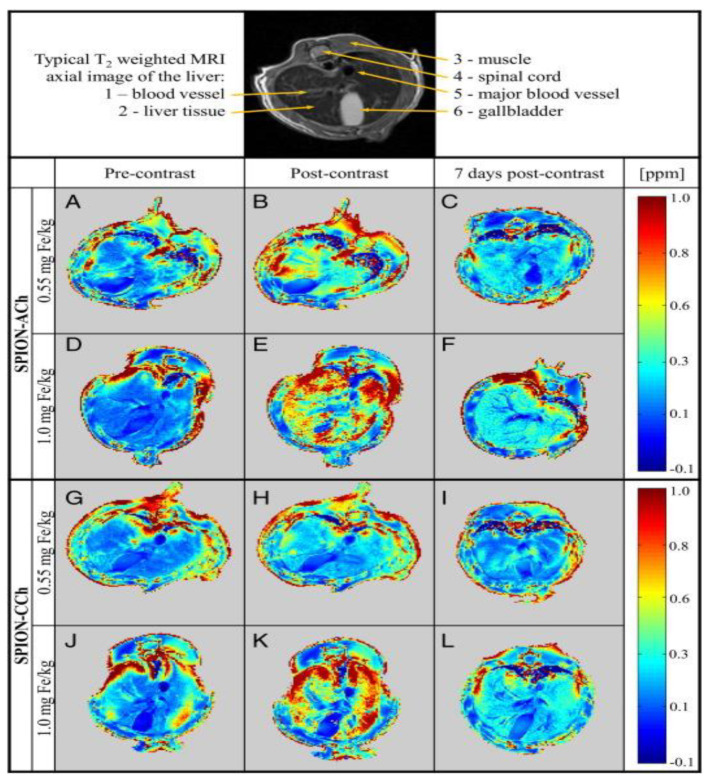
An overview of MNPs’ impact in MRI: The paramagnetic properties of various organs are detected effectively through MRI. The use of MNPs enhances image contrast significantly, but this improvement diminishes within a week, returning to baseline levels. Panels (**A**,**D**,**G**,**J**) are images taken at pre-contrast with different dosages of SPIONs. Panels (**B**,**E**,**H**,**K**) are images taken at post-contrast with different dosages of SPIONs. Panels (**C**,**F**,**I**,**L**) are images taken 7 days post-contrast with different dosages of SPIONs. Reprinted from [47], with permission from Elsevier.

Superparamagnetic nanoparticles are also utilized in analytical applications such as contrast enhancers in magnetic resonance imaging (MRI). Generally, the magnetic response of blood vessels comes from paramagnetic components like iron in hemoglobin and diamagnetic components from proteins made of basic elements like carbon, hydrogen, nitrogen, and oxygen. The imaging obtained from the particles is not visible enough. However, the magnetic signal from injected nanoparticles is far stronger, allowing better selectivity in biomedical applications [42]. Superparamagnetic nanoparticles create local variations in the magnetic field. When placed in a magnetic field, they induce changes in the magnetic environment surrounding nearby hydrogen protons in water molecules. The presence of these nanoparticles causes the spins of the hydrogen nuclei (protons) in water to lose their coherent alignment more quickly. As the magnetic environment fluctuates, the protons dephase faster, leading to a reduction in the relaxation time and allowing magnetization to be more spontaneous. This improves the visibility of specific tissues or abnormalities, aiding in precise diagnostics [43,44]. Figure 9 shows a comparison of MRI with and without bio-coated SPION being employed as contrasting agents. Post-contrast imaging reveals higher parts per million compared to imaging without a contrast agent. Simulations further indicate that a week later, the contrast diminishes, suggesting the degradation of MNPs in the biological environment and demonstrating their favorable biocompatibility. In general, MNP-based drug delivery systems offer a combination of controlled release, improved bioavailability, and reduced toxicity, making them a powerful tool for advancing personalized medicine and improving therapeutic outcomes in complex diseases such as cancer, neurological disorders, and infectious diseases [32,45,46].

## 3. Hydrogels

**Figure 10:**
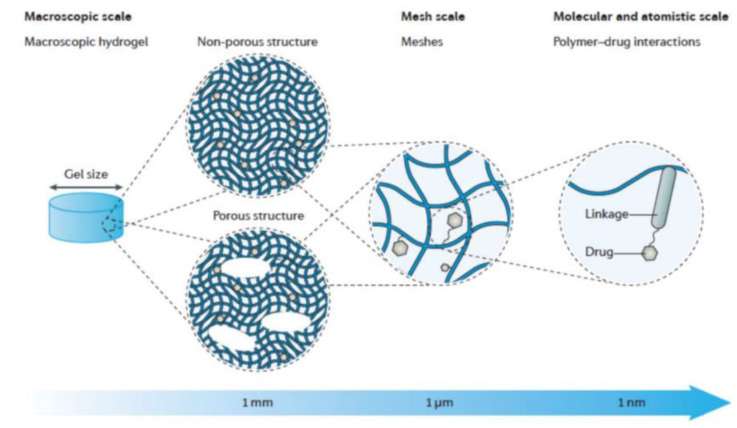
Structure of a hydrogel [50]. Used with permission from Springer Nature BV from Designing hydrogels for controlled drug delivery, J. Li and D. J. Mooney, Vol. 1, 2016; permission conveyed through Copyright Clearance Center, Inc.

**Figure 11:**
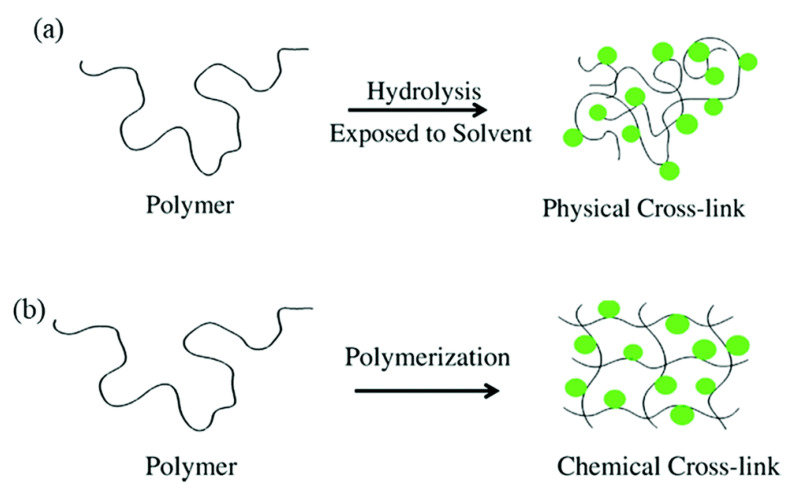
Schematic illustration of hydrogel fabrication. (**a**) Physical cross-linking. (**b**) Chemical crosslinking. Unedited image from P. Sikdar et al. [51] following the Creative Commons License, (http://creativecommons.org/licenses/by/4.0/).

Hydrogels are three-dimensional networks (Figure 10) capable of absorbing large amounts of water or biological fluids, allowing them to swell due to hydrophilic functional groups [48,49]. This property imparts flexibility and softness, and their design can involve chemical or physical crosslinking of natural or synthetic polymer chains (Figure 11) [49]. The network structure of hydrogels is formed through covalent or noncovalent interactions, including physical entanglements, hydrogen bonding, hydrophobic interactions, supramolecular interactions, electrostatic forces, and coordination interactions.

**Figure 12:**
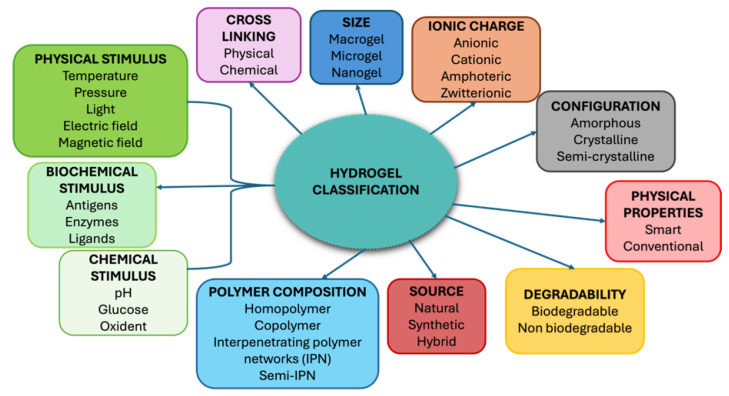
Summary of the Classifications of Hydrogels.

Natural and synthetic polymers have been explored to create hydrogels with desirable properties, including polypeptides, polysaccharides, DNA, polyacrylamide, and poly (vinyl alcohol) [52]. Hydrogels closely mimic living tissue, with high water content and tunable porosity, supporting applications in drug delivery. Hydrogels’ porosity, controlled by the degree of cross-linking, influences swelling, drug loading, and controlled release profiles, based on the diffusion coefficients of micro- and macromolecules [53]. Hydrogels are generally not recognized as foreign by the body’s immune system due to low interfacial tension with body fluids, which minimizes protein adsorption and cell adhesion [54]. The first hydrogel developed for biomaterials, poly-2-hydroxyethyl methacrylate (PHEMA), was created by Wichterle and Lim in 1960 for applications like eye enucleation fillers and contact lenses [55] and since then, their use in drug delivery and bioactive compound release has increased, though their use was often restricted to surface environments, such as for ocular applications or wound treatment [49]. Hydrogel properties, including swelling rate, stiffness, degradability, and mesh size, can be tailored by adjusting composition and reaction conditions. Biodegradable hydrogels can also be engineered to respond to hydrolytic, enzymatic, or environmental triggers such as pH, temperature, or electric fields [52,56,57]. Hydrogels experience significant volume or gel–sol phase transitions when exposed to specific physical and chemical stimuli. Physical stimuli include temperature, electric and magnetic fields, solvent composition, light intensity, and pressure, while chemical or biochemical stimuli encompass pH, ions, and specific chemical compositions. These transitions are often reversible, allowing hydrogels to return to their original state upon removal of the stimulus. Hydrogel response to stimuli depends on composition and cross-linking degree, with the magnitude of the response proportional to the applied stimulus [58]. Hydrogels can be classified based on different properties and Figure 12 provides a summary of the various classifications of hydrogels.

### 3.1. Fabrication of Magnetic Hydrogels

**Figure 13:**
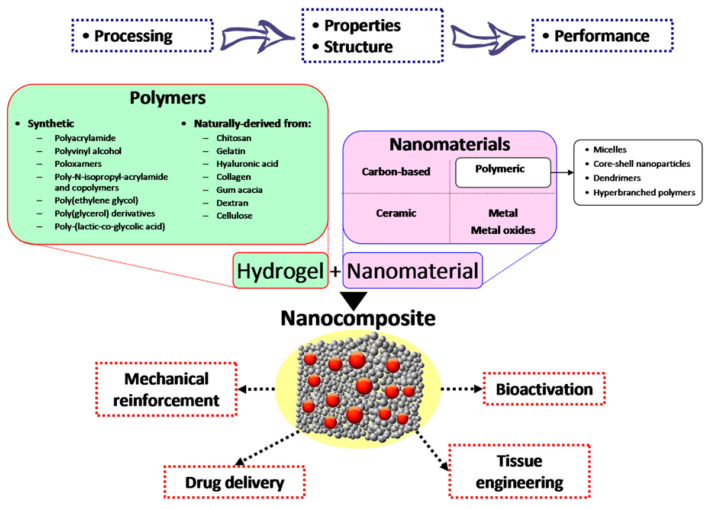
Illustration of nanocomposite hydrogels (NCHs) formed by embedding various nanosystems; carbon-based, polymeric, ceramic, and metal or metal-oxide nanoparticles, into hydrogel networks, resulting in enhanced properties and responsiveness to external stimuli. Unedited image from M. Biondi, A. Borzacchiello, L. Mayol, and L. Ambrosio [59] following the Creative Commons License, (http://creativecommons.org/licenses/by/4.0/).

Recent advancements in hydrogel technology have enabled the creation of nanocomposite hydrogels (NCHs), also known as nanocomposites, for biomedical applications. These NCHs consist of hydrated polymer networks with a 3D structure that swell in water and incorporate nanoparticles or nanostructures through covalent or non-covalent interactions [59]. Research is focused on integrating various nanoparticulate systems, including carbon-based, polymeric, ceramic, and metal or metal oxide nanoparticles, to produce NCHs, as seen in Figure 13 [60]. Among these, magnetic nanoparticles, typically composed of iron oxide, are embedded in the hydrogel matrix during synthesis, creating a hybrid system that enhances control over drug delivery. Magnetic hydrogels combine high drug-loading capacity and biocompatibility with magnetic responsiveness, generating localized heat under an external magnetic field and altering the matrix’s properties. This enables magnetic field-based control over drug release rates, offering precise dosing and targeted delivery, particularly valuable for cancer treatment [61,62].

**Figure 14:**
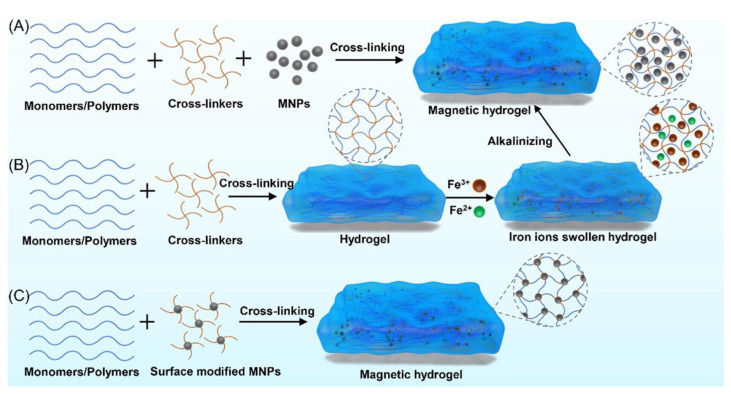
The construction of magnetic-responsive hydrogels for biomedical applications. Unedited image from S. Ganguly and S. Margel [63] following the Creative Commons License, (http://creativecommons.org/licenses/by/4.0/).

Blending is the most common and straightforward method for fabricating magnetic composite hydrogels. Fe_3_O_4_ is the most frequently used magnetic nanoparticle (MNP) blended with polymer systems to create these hydrogels. This process involves two main steps: first, MNPs are synthesized and stored in an aqueous oil phase to prevent oxidation. Next, the MNPs are mixed with hydrogel precursor materials before the cross-linking of the polymer matrix occurs. The mixing technique is a low-energy, rapid method for developing composite hydrogels. This approach also allows for the incorporation of MNPs with varying particle size distributions into hydrogel matrices (Figure 14) [63].

The synthesis of in situ MNP-based composite hydrogels offers greater control over particle size and hydrogel architecture [64]. In this method, MNPs are formed within the gel matrices during the gelation process. Initially, precursor metal ions for the MNPs are dispersed into the hydrogel precursor materials, particularly the monomers [65]. As gelation occurs, an insoluble solid gel mass is produced, which is subsequently treated with an alkali solution to generate MNPs within the hydrogel matrix [63,66].

The two previously discussed approaches share a common feature: both methods involve non-covalent entrapment of MNPs within gel matrices. In contrast, the grafting-onto technique chemically modifies MNPs to enhance compatibility with the polymer system. Here, MNPs are chemically bonded to hydrogel polymer chains, providing greater stability compared to physical or simple blending methods. In this method, the modified MNPs act as cross-linkers within the hydrogel system, integrating directly into the network [63].

### 3.2. Applications of Magnetic Hydrogels in Cancer Treatment

**Figure 15:**
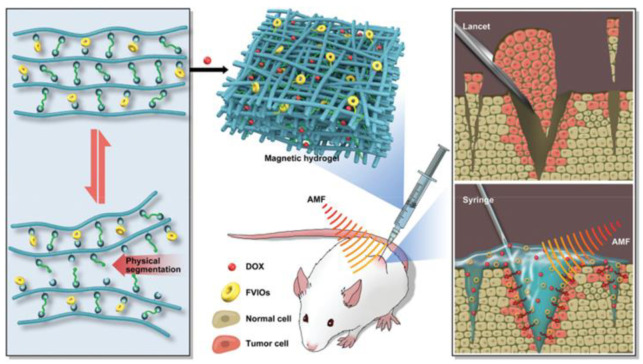
FVIO-functionalized magnetic hydrogel with optimal adaptive functions for breast cancer postoperative recurrence prevention. Unedited image from F. Gao et al. [67] following the Creative Commons License, (http://creativecommons.org/licenses/by/4.0/).

In cancer treatment, localized drug release is essential to minimize systemic side effects. Magnetic hydrogels can deliver chemotherapy drugs directly to the tumor site, releasing them upon magnetic activation. This targeted approach allows higher drug concentrations to reach the tumor while reducing exposure to healthy tissues, improving the therapeutic outcome and reducing adverse effects. Gao et al. developed a novel magnetic hydrogel using difunctional telechelic polyethylene glycol (DT-PEG), glycol-chitosan, and ferromagnetic vortex-domain iron oxide (FVIO) to enable efficient cancer treatment [67]. This hydrogel, with a low FVIO concentration, demonstrated strong inductive heating, sustained drug release, and pH-responsiveness. In vivo studies showed effective chemo-thermal therapy in mice, reducing tumor size, preventing local recurrence, and enhancing antitumor effects while minimizing side effects on healthy tissue (Figure 15) [67].

An injectable, biodegradable, and thermosensitive SPION-loaded nanocapsule hydrogel (SPION-NH) was developed by Zhang et al. for multiple magnetic hyperthermia therapy (MHT) at moderate temperatures and extended MRI monitoring from a single injection [68]. Using a sol–gel phase transition at body temperature, the SPION-loaded solution transforms into a hydrogel upon injection, enabling long-term SPION retention in tumors and effective multiple MHT. Four MHT cycles demonstrated strong anti-tumor effects in a mouse xenograft model with no damage to surrounding tissues, while T_2_-weighted MRI provided three weeks of therapeutic monitoring. This thermosensitive SPION-NH system shows promise as a safe, long-term theranostic platform for clinical trials, either alone or combined with chemotherapy and radiotherapy for enhanced cancer therapy [68].

In a research paper made by Panja et al., they introduced a smart, light-responsive nanogel designed for precise, on-demand cancer therapy [69]. Constructed from a branched pentaerythritol poly(caprolactone)-b-poly(acrylic acid) (PE-PCL-b-PAA) copolymer crosslinked with minimal ferric ions (Fe^3+^), the nanogel enabled controlled drug release when activated by light, achieving up to 85% doxorubicin (DOX) release within 120 min. In vitro and in vivo studies, specifically in a glioma model, demonstrated high cancer cell uptake and significant tumor inhibition (91%) with minimal toxicity, underscoring the nanogel’s potential for effective and targeted cancer treatment [69].

Qian et al. developed an injectable magnetic hydrogel by combining iron oxide nanocubes with silk fibroin, using sonication to aid the process [70]. The resulting ferrimagnetic silk fibroin hydrogel exhibited shear-thinning properties, allowing it to be easily injected into deep tumor sites. Under an alternating magnetic field (AMF) of 30 kA/m and 312 kHz for 15 min, the hydrogel demonstrated effective remote heating and significant anti-tumor effects. Additionally, it was observed that the ferrimagnetic hydrogel induced blood vessel occlusion, suggesting its potential to block the tumor’s blood supply [71].

Yan et al. also developed a multifunctional hybrid hydrogel (NDP-FG) along with a multidisciplinary approach for hepatocellular carcinoma (HCC) therapy [72]. During hepatectomy, the NDP-FG hydrogel demonstrated highly effective intraoperative hemostasis and helped prevent tumor recurrence. Its stable vascular embolization capability suggests that the NDP-FG hydrogel holds significant promise for HCC treatment through transarterial embolization. This compatibility with both postoperative and transarterial embolization therapies highlights the potential of NDP-FG to advance clinical strategies and materials for treating liver cancer and other solid tumors [72].

**Figure 16:**
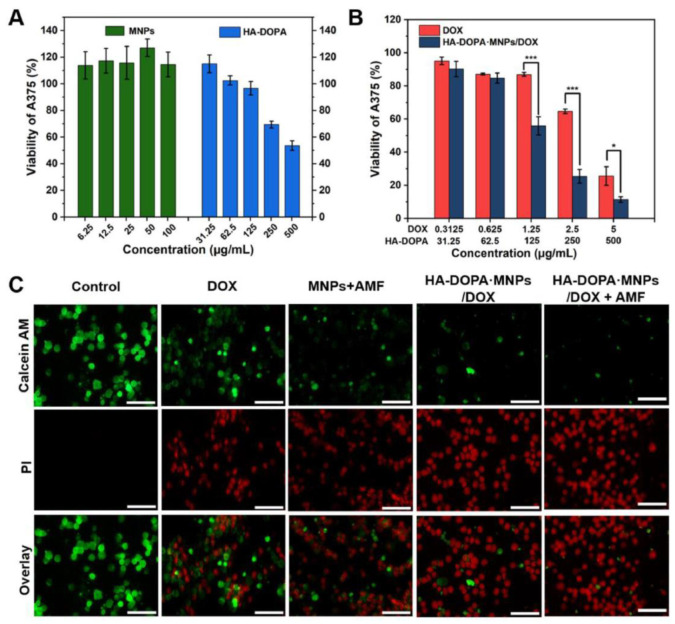
(**A**) Viability of human melanoma cells (A375) following 24-h treatment with magnetic nanoparticles (MNPs) and an injectable hydrogel composed of dopamine-conjugated hyaluronan (HA-DOPA). (**B**) Viability of A375 cells after 24 h of exposure to the anti-cancer drug doxorubicin (DOX) and a DOX-loaded hydrogel. (**C**) Fluorescence images of A375 cells stained with calcein AM (green, live cells) and propidium iodide (PI, red, dead cells) after different treatments, demonstrating the effects of magnetic fields and anti-cancer drugs on cell viability. Scale bar: 100 µm. (Mean ± SD, n = 5, * *p* < 0.05, *** *p* < 0.001). Reprinted from [73], with permission from Elsevier.

In a related study, Dai et al. developed a nanocomposite hydrogel synthesized using a dopamine-Fe^3+^ complex reinforced with magnetic nanoparticles (MNPs) [73]. The inclusion of MNPs significantly enhanced the hydrogel’s shear modulus. Combining hyperthermia with targeted drug delivery, the hydrogel demonstrated improved efficacy against cancer. Controlled by an external magnetic field, it could be heated non-contact, releasing the anti-cancer drug (doxorubicin, DOX) in pulses when the alternating magnetic field (AMF) was activated and reverting to slow release when turned off. In vivo, the DOX-loaded composite hydrogel exhibited a longer retention time than both the DOX-loaded gel and DOX solution. The study also proposed that single-modal treatment, using either the drug or hyperthermia alone, would be less effective than the combined approach. Cell assays indicated that the simultaneous application of magnetic fields and anti-cancer drugs led to higher rates of cell death compared to using either treatment independently (Figure 16) [73].

### 3.3. Limitations and Future Outlook

A significant challenge with hydrogels is their inherently hydrophilic nature, while approximately 40% of marketed drugs and nearly 90% of therapeutic compounds in development exhibit poor water solubility [74]. This mismatch can lead to limited drug dispersion and loading within the hydrogel matrix, resulting in suboptimal release profiles. Hydrophobic drugs often display low dissolution rates and poor bioavailability, necessitating higher doses to reach therapeutic concentrations at the target site [75]. While magnetic hydrogels offer the promising ability to remotely control pulsatile drug release via an external magnetic field, challenges remain in effectively targeting deep tissue regions, as the targeting efficiency diminishes with increased distance between the tumor and the magnets. Additionally, considerations of cytotoxicity, biodegradability, and the long-term fate of embedded magnetic nanoparticles (MNPs) in vivo are critical. Since there is no universal criterion to predict the clearance of MNPs due to their varied physicochemical properties, a two-step clearance process is essential. First, as hydrogels biodegrade and cells produce extracellular matrix, embedded MNPs can be released [76]. Second, these MNPs must be efficiently eliminated from the body. The FDA has approved certain MNPs, such as those used in magnetic resonance imaging (MRI), and they are rapidly cleared through the body’s natural iron metabolism pathways, primarily through the reticuloendothelial system (RES), especially the liver [77]. MNP elimination also depends on particle size; for example, particles under 5.5 nm are quickly cleared renally, particles up to 500 nm through the liver, and particles up to 5 μm via lymphatic drainage [77,78]. Further investigation into the biodistribution and elimination of other metallic (e.g., cobalt, nickel) and bimetallic (e.g., platinum) MNPs in vivo will be vital to expanding clinical applications for cancer therapy.

## 4. Liposomes, Micelles, Niosomes, and Exosomes

**Figure 17:**
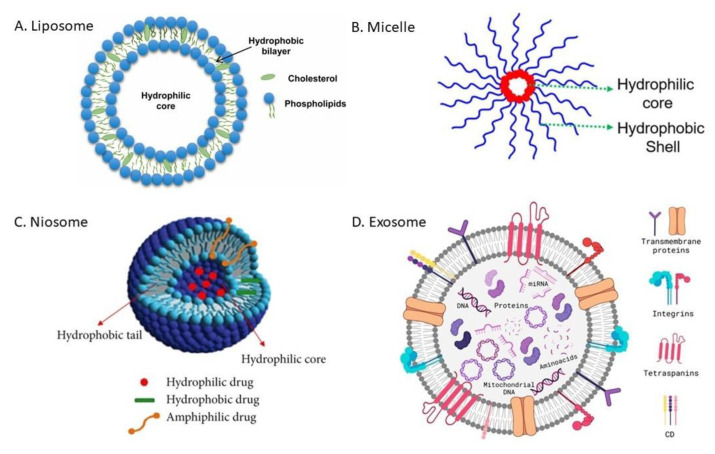
Schematic diagram of a general structure of: (**A**) liposome [86], (**B**) micelle [87], (**C**) noisome [88], and (**D**) exosome [89]. Image (**A**) adapted from H. Nsairat et al. [86], image (**B**) adapted from S. Perumal et al. [87], image (**C**) adapted from M. Gharbavi et al. [88], and image (**D**) adapted from M. A. Tienda-Vázquez et al. [89], all following the Creative Commons License, (http://creativecommons.org/licenses/by/4.0/).

MNPs represent a powerful tool in cancer drug delivery, referring to the possibility of targeted delivery and controlled release of therapeutic agents [20,79]. When complexed with nanocarriers such as liposomes, micelles, niosomes, and exosomes (Figure 17), MNPs will provide the magnetic guidance of drugs toward specific tumor sites and release them under controlled conditions [80,81]. This approach of treatment increases its efficacy by concentrating therapeutic agents on cancerous tissues, reducing off-target effects and systemic toxicity associated with conventional therapies [82,83]. Magnetic responsiveness from these MNP-carrier hybrids allows drug release in conditions such as magnetic hyperthermia, where the process can be locally heated to induce release with the application of an external magnetic field for precise control over temporal and spatial specifications of drug release [84,85].

### 4.1. Liposomes

**Figure 18:**
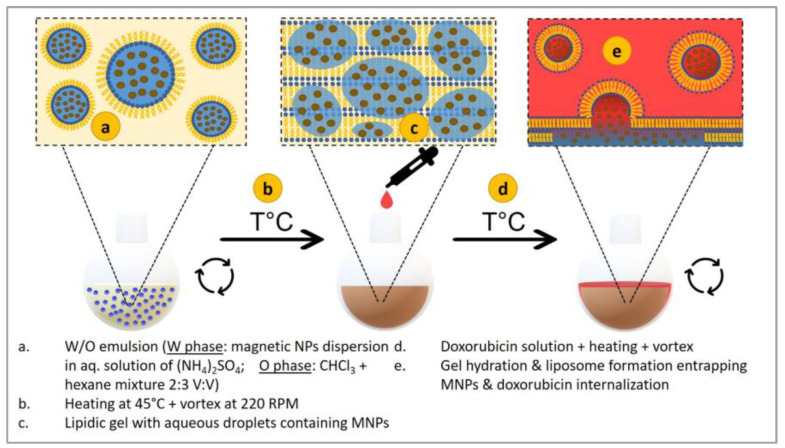
Schematic presentation of synthesizing targeted magneto-liposomes loaded with DOX under an applied magnetic field. Unedited image from S. Nitica et al. [91] following the Creative Commons License, (http://creativecommons.org/licenses/by/4.0/).

Liposomes (Figure 17A) are spherical vesicles formed by phospholipid bilayers, and their structural versatility and biocompatibility have motivated their wide use in drug delivery [90]. The embedment of MNPs within liposomes yields magneto-liposomes, which have shown great promise in cancer treatment. Extensive research involving this hybrid system has been conducted, especially toward targeting elusive tumors [91,92]. Shevtsov et al., (2015) demonstrated the efficacy of magneto-liposomes in targeting glioblastoma cells, where an externally applied magnetic field could effectively direct such carriers to the tumor site, thereby enhancing drug accumulation and therapeutic benefit [92]. The work emphasized the role that MNPs could play in addressing the challenges posed by precise drug localization to complex tumor geometries [93]. In this respect, similar studies conducted by Nitica et al., (2022) showed that magneto-liposomes loaded with DOX could be targeted, under an applied magnetic field, toward breast cancer tumors in animal models (Figure 18) [91]. This resulted in a significant increase in drug intake within the tumor when the drug was administered as free compared to magneto-liposomes. The application of an external magnetic field basically does the targeting by magneto-liposomes. MNPs can act as a “homing” mechanism, which delivers the liposomes to the tumor site [20,94].

Acharya et al., (2020) studied magnetic nanoparticle-assisted drug release from liposomes to enhance the utility of liposomal drug delivery systems [95]. They developed a novel approach using gold–thiol chemistry to integrate magnetic nanoparticles with liposomes. Gold-coated magnetic nanoparticles encapsulated thiolated liposomes externally, eliminating competition for internal space and allowing full utilization for drug loading. The liposome formulation included DPPC, DSPC, and either cholesterol or cholesterol-PEG-SH. Permeability assays and electron microscopy confirmed efficient coupling of liposomes and nanoparticles without compromising functionality. Under pulsed magnetic fields, up to 20% of the encapsulated drug was released, with efficiency comparable to previous magneto-liposome studies. Release efficiency also varied with different dilution media due to osmotic pressure effects on stability [95].

**Figure 19:**
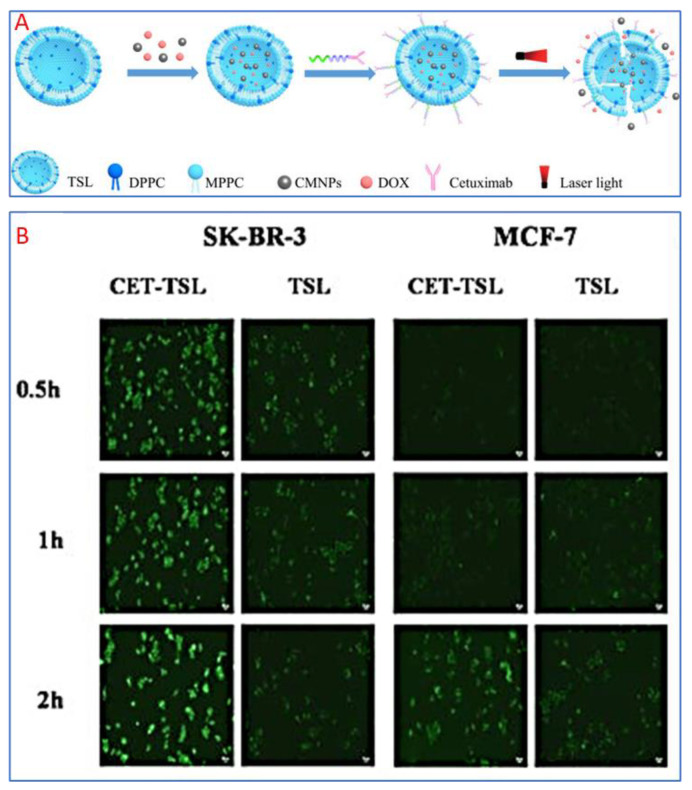
(**A**) is graphical illustration of NIR-triggered DOX release from CET-DOX-CMNP-TSLs. (**B**) is fluorescence microscopy of cellular uptake of TSLs coated with and without CET by SKBR-3 and MCF-7 cells. Unedited images from B. Dorjsuren et al. [99] following the Creative Commons License, (http://creativecommons.org/licenses/by/4.0/).

The phospholipid bilayer structure in liposomes provides ease and flexibility in encapsulating drugs of both hydrophilic and lipophilic nature and delivering a wide range of chemotherapeutic agents [96,97,98]. Dorjsuren et al., (2020) developed a liposome-based nanoparticle drug delivery system to enhance cancer chemotherapy by targeting tumor cells specifically and minimizing systemic toxicity (Figure 19A) [99]. They engineered citric acid-coated iron oxide magnetic nanoparticles (CMNPs) and incorporated them into thermo-sensitive liposomes (TSLs) loaded with the chemotherapeutic agent DOX. These TSLs were further coated with cetuximab (CET), an antibody targeting epidermal growth factor receptor (EGFR)-expressing breast cancer cells. The CET-DOX-CMNP-TSLs demonstrated increased cellular uptake and enhanced therapeutic efficacy (Figure 19B). Activating by near-infrared (NIR) laser irradiation leads to effective photo-thermal therapy and significant reductions in cancer cell viability. In vivo studies showed that NIR irradiation raised tumor temperatures to 44.7 °C and 48.7 °C, effectively contributing to tumor reduction. Hemolysis tests confirmed the safety of these nanocarriers for systemic use, suggesting that combining photo-thermal therapy with targeted chemotherapy via thermo-sensitive nanocarriers is a promising approach for treating breast cancer.

In addition to passive targeting mechanisms like EPR effect, MNP-enhanced liposomes provide a layer of active targeting through external magnetic guidance [100,101]. This has been particularly beneficial in addressing the heterogeneity of tumor vasculature, which can limit the efficacy of passive targeting alone [101,102]. For instance, Moloney et al., (2023) explored the use of magneto-liposomes in liver cancer treatment, where the combination of magnetic targeting and liposome encapsulation improved drug delivery within the highly vascularized pancreatic tumor environment [103]. This study highlighted how magnetically guided liposomes could overcome the uneven distribution of drugs caused by inconsistent vascular permeability in tumors.

### 4.2. Micelles

Micelles (Figure 17B), nanoscale aggregates formed through the self-assembly of amphiphilic molecules, are another potential platform for MNP loading. Micelles are composed of a hydrophobic center and hydrophilic outer shell, which make them ideal to contain hydrophobic drugs [104,105,106]. Magnetic Micelles refer to the MNPS that are incorporated in micelles, which are further directed by magnetic fields at the tumor sites [107]. Zhang et al., (2017) studied magnetic micelles administration of paclitaxel (the most widely used antitumor drug) in breast cancer tumors [108]. Using magnetic guidance, the micelles were shown to accumulate more paclitaxel in the tumor, a finding that led to a better decrease in the tumor size compared to non-magnetic micelles. Consequently, they concluded that this effect highlights the potential of using MNPs to enhance the therapeutic index of micelle-encapsulated therapeutic agents for cancer therapy.

**Figure 20:**
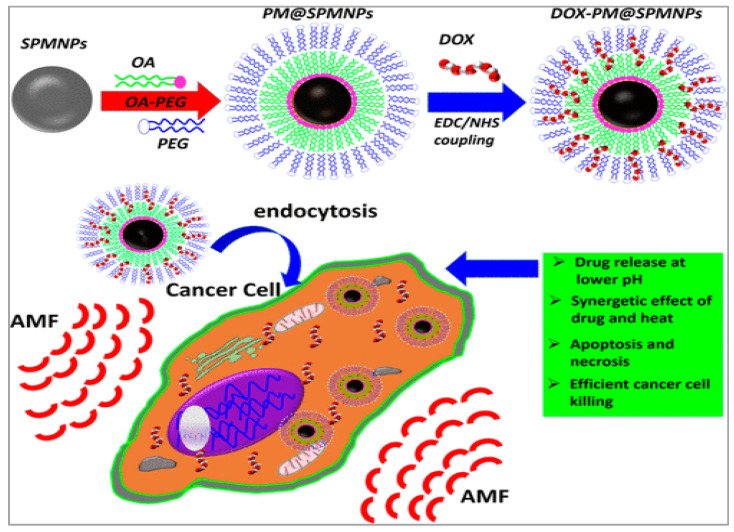
Mechanism of anti-cancer drug (DOX) loading into polymer-coated SPMNPs and the approach for cancer cell destruction through combined therapy using an alternating magnetic field (AMF). Reprinted with permission from [109]. Copyright 2017 American Chemical Society.

**Figure 21:**
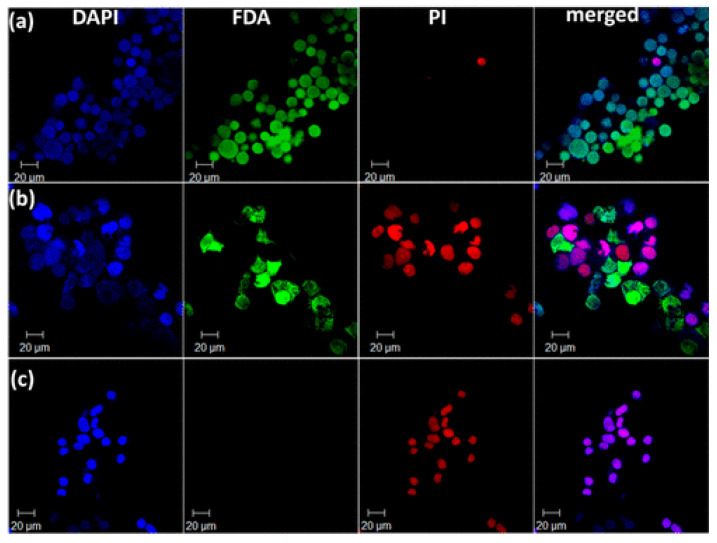
In vitro analysis by confocal microscopy images of untreated (**a**) and MCF7 cells treated with MFH generated by DOX-PM@SPMNPs for (**b**) 30 min and (**c**) 60 min, followed by staining with DAPI, PI, and FDA after 24 h of incubation (scale bar: 20 μm). Background fluorescence from DOX was eliminated using LSM imaging software. Reprinted with permission from [109]. Copyright 2017 American Chemical Society.

The controlled release of drugs from magnetic micelles is often achieved through magnetic hyperthermia. This approach utilizes the heat generated by MNPs under an alternating magnetic field to destabilize the micelle structure, releasing the encapsulated drug. Thorat et al., (2017) explored a combined chemotherapy and nanoparticle-based hyperthermia approach to tackle drug resistance in cancer [109]. They functionalized superparamagnetic La_0.7_Sr_0.3_MnO_3_ nanoparticles (SPMNPs) with an oleic acid and polyethylene glycol (PEG) micelle structure, loading them with DOX at a high capacity (Figure 20). In vitro, DOX-loaded SPMNPs achieved an 89% cancer cell death rate, surpassing free DOX, and induced 80% cell extinction within 30 min via hyperthermia (Figure 21). In vivo studies in mice showed favorable biodistribution, indicating that these micellar SPMNPs are promising candidates for combined chemotherapy-hyperthermia cancer therapy.

### 4.3. Niosomes

**Figure 22:**
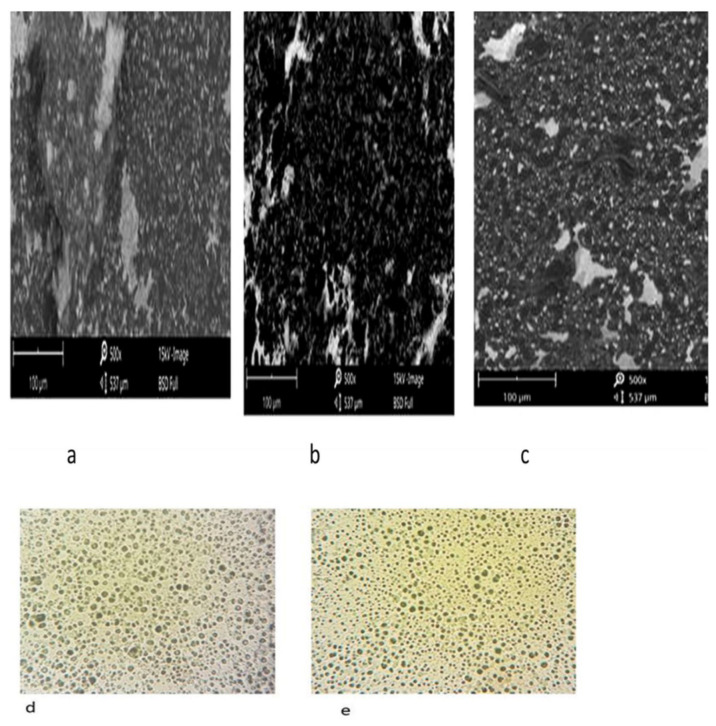
Scanning electron microscopy images show the morphology of Span 60-based niosomes prepared using (**a**) THF, (**b**) EVP/SON, and (**c**) RPE (magnification ×500). Additionally, photomicrographs display (**d**) Span 60-based niosomes (TFH) and (**e**) Tween 60-based niosomes (TFH) at ×400 magnification. Reprinted from [114], with permission from Elsevier [114].

Niosomes (Figure 17C) are stable and cheaper carrier matrices and have structures similar to liposomes, except that the vesicle is made from non-ionic surfactants [110,111]. Magnetic niosomes combine the stability and biocompatibility of niosomes in the way MNPs can be directed or targeted to a region of interest, providing an excellent platform for cancer treatment applications of drug delivery [112,113]. Ugorji et al., (2022) studied the delivery of 5-fluorouracil to colon cancer cells in vitro by magnetic niosomes, showing that an external magnetic field enhanced drug uptake by tumor cells [114]. They tested different preparation methods such as thin film hydration (TFH), reverse phase evaporation (RPE), evaporation/sonication (EVP/SON), and ethanol injection method (EIM) with Tween 60 and Span 60 surfactants, finding that TFH produced smaller particles with higher drug entrapment, especially with Tween-based niosomes (Figure 22). Niosomes have a potential application due to their physical stability in a biological environment, sustained drug release, and resistance towards pre-leakage of drug which is truly important to reach the effective concentration at the tumor site. The results of drug delivery by this application of magnetic fields to induce the guidance of niosomes and magnetic control during niosome release suggest an ability to reduce the side effects of conventional drug treatment and, therefore, improve patient outcomes [110,113]. A study by Davarpanah et al., (2018) utilized magnetic niosomes encapsulated with carboplatin against MCF-7, a breast cancer cell line, treatment in vitro and found that magnetically guided niosomes promote the accumulation of drugs within cancer cells while sparing healthy cells [115]. This result also highlighted how combining MNPs with niosomes may provide spatially targeted drug delivery to sensitive organs, where off-target toxic effects may result in significantly adverse consequences.

### 4.4. Exosomes

**Figure 23:**
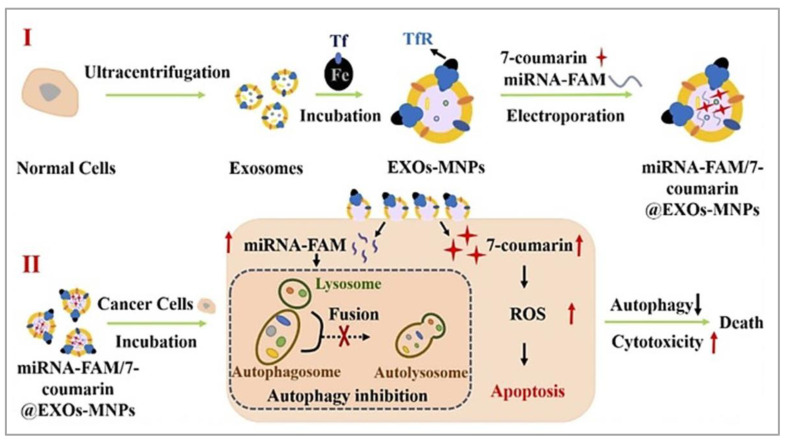
A schematic diagram illustrating (**I**) the preparation process and (**II**) the synergistic cancer therapy mechanism of the miRNA-FAM/7-coumarin@EXOs-MNPs drug delivery system. Unedited image from X. Hua et al. [119] following the Creative Commons License, (http://creativecommons.org/licenses/by/4.0/).

Exosomes (Figure 17D) are natural-derived extracellular vesicles and provide inherent biocompatibility, sized between 30 and 200 nm, which can pass biological barriers. This feature makes them a good option for cancer therapy. Exosomes have specialized surface molecules, like immune regulators and membrane proteins, that help them recognize and attach to specific target cells or avoid others. This selective ability makes them highly effective messengers, delivering biomolecules directly to the cells that need them and enabling precise communication between cells. The natural origin of exosomes helps evade immune detection, prolonging circulation time and allowing for extended drug action in the tumor microenvironment. Exosomes have been studied for their potential in MNP-based drug delivery systems, and their targeting capabilities, when combined with MNPs, provide a dual mechanism of action. Exosomes can be functionalized with surface ligands that bind precisely to tumor receptors, and the magnetic field helps in concentrating the exosomes at the tumor site [89,116,117,118]. Hua et al., (2024) developed an advanced exosome-based drug delivery system for ovarian cancer [118]. They utilized exosomes as natural biocompatible vesicles and immune evasion for targeted therapy [119]. By attaching MNPs to the exosomes for easier separation, they loaded two agents, including miRNA-FAM for autophagy inhibition and 7-coumarin for apoptosis induction into the exosome-MNP complex (Figure 23). This dual-drug system showed improved cancer cell targeting and penetration, with the combined action significantly enhancing therapeutic efficacy. This innovative approach offers promising potential for more effective, targeted cancer treatments in future clinical applications.

Raymond et al., (2016) demonstrated the role of exosomes containing the HIV protein Nef in disrupting the blood–brain barrier (BBB) and contributing to HIV-associated neuropathogenesis [120]. Specifically, it was found that only nef-transfected microglia release exosomes carrying Nef (exNef), which significantly impaired BBB integrity by reducing tight junction proteins and increasing permeability. To counteract these effects, the researchers employed MNPs to deliver Nef peptides across the BBB. These peptides, targeting the Nef myristoylation site, effectively inhibited exNef release from microglia and reduced its disruptive impact on the BBB. This exosome-targeted, MNP-based approach presents a promising strategy for mitigating HIV-related neuroimmune damage by blocking exNef-mediated effects, showing potential for future therapeutic applications [120].

### 4.5. Challenges

While the use of MNPs with these nanocarriers offers numerous advantages, challenges remain in optimizing their delivery and stability. For instance, maintaining the stability of magneto-liposomes and magnetic niosomes in physiological conditions is essential for effective in vivo applications. Researchers have explored PEGylation and other surface modifications to enhance the stability and circulation time of these carriers, as seen in studies with PEGylated magneto-liposomes in ovarian cancer models. These modifications aim to reduce immune recognition and prolong the presence of the carriers in the bloodstream, ensuring more efficient tumor targeting. The scalability of MNP-carrier hybrid systems also presents a challenge. Although these systems show progress in preclinical studies, applying them to clinical settings requires much testing to ensure consistency, reproducibility, and safety. The potential for MNP accumulation in non-target tissues must be addressed. Strategies such as biodegradable MNPs are being explored to minimize long-term toxicity and enhance the biocompatibility of these systems.

## 5. Dendrimers

The versatility of lipid-based nanocarriers, including exosomes, micelles, and liposomes, demonstrates their critical role in advancing MNP-mediated drug delivery systems. These systems leverage lipid structures to achieve biocompatibility, extended circulation, and precise targeting capabilities, creating synergistic opportunities when coupled with MNPs. However, the challenges of stability, scalability, and targeted delivery underscore the need for innovative solutions to overcome these limitations. One such solution lies in the development of dendrimers—highly branched, nanoscale polymers [121]. These unique structures offer unmatched functional versatility, enabling precise surface modification and enhanced compatibility with MNPs. Dendrimer functionalized magnetic nanoparticles (DFMNPs) not only address the shortcomings of lipid-based systems but also introduce additional benefits, such as high drug-loading capacity and multi-modal targeting, marking a pivotal evolution in nanocarrier design for cancer therapy [122].

**Figure 24:**
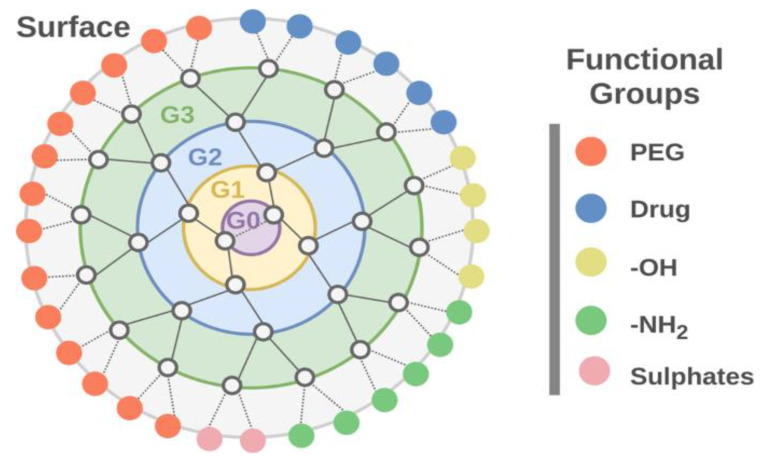
Basic structure of a dendrimer showing sequential layering of monomeric units around a central core (G0). A dendrimer may be composed of any monomeric unit provided it has at least 2 functional groups available to build additional generations. Surface functional groups depicted as circles. Unedited image from L. M. Kaminskas, D. E. V. Pires, and D. B. Ascher [123] following the Creative Commons License, (http://creativecommons.org/licenses/by/4.0/).

Dendrimers are highly branched, three-dimensional polymers characterized by their monodisperse and globular architecture. Their distinct structure comprises a central core, interior layers (or generations), and terminal functional groups, as illustrated in Figure 24. The diagram illustrates the hierarchical organization of dendrimer generations (G0–G3) and their associated functional groups on the surface. The abundance of terminal functional groups provides numerous reactive sites for attaching therapeutic agents, imaging molecules, or other functional groups, making dendrimers highly suitable for drug delivery applications (Figure 24) [121,123].

The synthesis of dendrimers can be broadly categorized into divergent and convergent methods, both of which allow for the systematic addition of monomers to achieve a controlled and well-defined structure [122]. In the divergent approach, growth starts from the core and progresses outward by adding monomer units step-by-step, whereas the convergent method involves the synthesis of dendrimer segments (dendrons) that are subsequently joined to the core. Before dendrimer attachment, though, magnetic nanoparticles typically require surface modification to provide suitable anchoring points. Common approaches include silane coupling, carboxylate modification, and phosphonate anchoring. Silane coupling involves modification with aminosilanes like (3-aminopropyl)triethoxysilane (APTES), which provides primary amine groups for dendrimer attachment [124]. The silane coating also helps protect the magnetic core from oxidation while providing colloidal stability. Carboxylate modification involves surface functionalization with carboxylic acid groups through citric acid or similar molecules, enabling dendrimer attachment via amide bond formation [125]. Phosphonate anchoring uses phosphonate groups to provide strong binding to the metal oxide surface through mono-, bi-, or tridentate coordination, offering enhanced stability compared to other anchoring methods [126].

Dendrimer conjugation to magnetic nanoparticles can be achieved through a variety of chemical strategies, including covalent and non-covalent approaches. Covalent conjugation typically involves forming stable chemical bonds between functional groups on the dendrimer and reactive groups on the MNP surface. For instance, carbodiimide chemistry is often used to link amine groups on dendrimers to carboxyl groups on the nanoparticle surface, resulting in a stable amide bond [127]. Another commonly used method is the formation of thiol-gold bonds, particularly when gold-coated magnetic nanoparticles are employed [128].

Non-covalent conjugation methods, such as electrostatic interactions, hydrophobic interactions, and hydrogen bonding, are also widely used. Electrostatic conjugation relies on the attraction between oppositely charged groups on dendrimers and MNPs, providing a simpler, though less stable, means of functionalization. This approach allows for easier modification and reversibility, which can be advantageous in certain therapeutic contexts where controlled release is desired [129].

The choice of conjugation method significantly affects the properties of the resulting dendrimer-functionalized MNPs, including stability, drug release profile, and targeting efficiency. Covalent bonds generally provide greater stability in physiological conditions, while non-covalent interactions offer flexibility and adaptability for applications where dynamic binding is beneficial.

Drug loading into dendrimer-functionalized magnetic nanoparticle systems can be achieved through multiple mechanisms, taking advantage of both the dendritic architecture and the magnetic core. The loading strategies can be broadly categorized into physical encapsulation within dendrimer cavities, chemical conjugation to surface groups, and combinations thereof [130].

**Figure 25:**
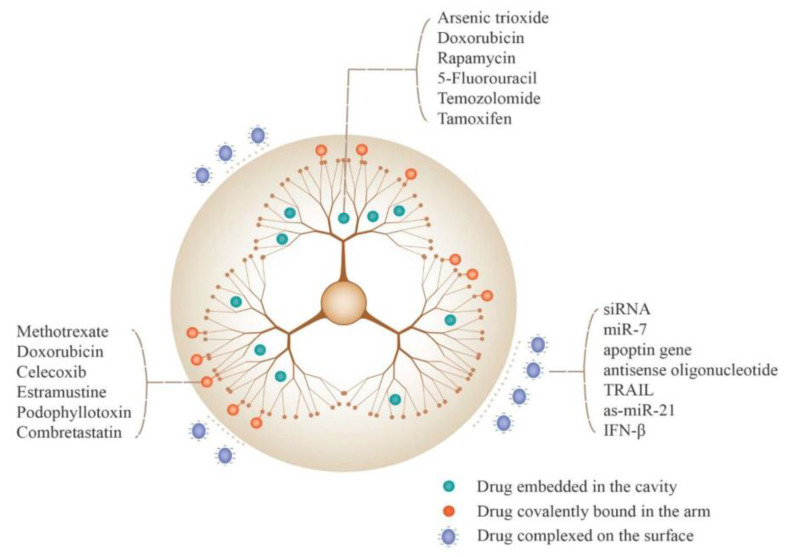
Methods for incorporating drugs to PAMAM-based DDSs and examples of drugs that can be loaded using various methods. Unedited image from X. Li, W. Ta, R. Hua, J. Song, and W. Lu [131] following the Creative Commons License, (http://creativecommons.org/licenses/by/4.0/).

Physical encapsulation exploits the unique architecture of dendrimers, which provides internal cavities capable of hosting drug molecules through various non-covalent interactions. These interactions include hydrophobic associations, hydrogen bonding, and electrostatic attractions. For instance, doxorubicin, a commonly used anti-cancer drug, can be efficiently encapsulated within PAMAM dendrimers through both hydrophobic interactions with the dendrimer interior and ionic interactions with internal tertiary amines (Figure 25) [125]. The loading capacity depends significantly on dendrimer generation, with higher generations typically offering increased drug loading due to their larger internal volumes and more numerous binding sites.

Chemical conjugation provides an alternative approach where drugs are covalently attached to functional groups on the dendrimer surface. This method offers more stable drug attachment and precise control over drug loading ratios. Common conjugation strategies include amide bond formation between drug molecules and surface amine groups, or ester linkages that can be hydrolyzed under specific conditions. For example, methotrexate has been successfully conjugated to dendrimer-MNP systems through carbodiimide chemistry, forming biodegradable bonds that enable controlled release in tumor environments [132].

The pH-responsive nature of many dendrimer systems plays a crucial role in drug loading and release. At physiological pH (7.4), the internal tertiary amines of PAMAM dendrimers remain largely unprotonated, allowing for efficient drug encapsulation. However, in the acidic environment of tumor tissues (pH 6.5) or endosomal compartments (pH 5.0–5.5), these amines become protonated, leading to conformational changes that facilitate drug release [133]. This pH-dependent behavior enables selective drug release at tumor sites while minimizing premature release during circulation.

Dual loading strategies that combine physical encapsulation and chemical conjugation have also been explored to enhance therapeutic efficacy. These approaches allow for the simultaneous delivery of multiple drugs with different release kinetics, enabling combination therapy approaches. For instance, some systems have demonstrated successful co-delivery of hydrophobic drugs encapsulated within dendrimer cavities alongside surface-conjugated targeting molecules or imaging agents [134].

The magnetic core provides additional opportunities for triggered drug release through application of alternating magnetic fields. Local hyperthermia generated by the magnetic core can induce conformational changes in the dendrimer structure or break thermosensitive bonds, providing an external trigger for controlled drug release. This magnetic responsiveness, combined with the inherent pH-sensitivity of dendrimers, enables multi-responsive drug delivery systems that can be precisely controlled both spatially and temporally.

The functional groups on the dendrimer surface can be modified to target specific receptors on cancer cells, improving the targeting efficiency of the drug delivery system [128]. For example, folic acid, a well-known targeting ligand, can be conjugated to dendrimers to target folate receptors, which are overexpressed in many cancer cells [128].

Dendrimers’ ability to encapsulate small drug molecules in their internal cavities or bind them to surface groups, enabling high drug payloads and controlled release, is primarily influenced by their unique branched structure, which allows for multiple interaction sites [135]. Unlike larger systems such as hydrogels, which can also encapsulate drugs, the nanoscale size of dendrimers allows for enhanced permeability and retention at target sites, making them particularly advantageous for targeted drug delivery. In combination with MNPs, dendrimers can enhance the therapeutic efficacy of drug-loaded systems by providing both stabilization and targeting functionality.

A notable example involves the use of doxorubicin, an anti-cancer drug, conjugated to dendrimer-functionalized MNPs. Doxorubicin-loaded PAMAM-coated MNPs demonstrated improved cellular uptake, enhanced cytotoxicity against cancer cells, and the ability to be directed to the tumor site using an external magnetic field [136,137]. Additionally, the controlled release of doxorubicin from the dendrimer structure resulted in a sustained therapeutic effect, which is critical for minimizing side effects associated with conventional chemotherapy.

The effectiveness of DFMNPs in cancer therapy relies heavily on their ability to selectively accumulate at tumor sites. These systems can exploit multiple targeting mechanisms simultaneously, combining passive targeting through the enhanced permeability and retention (EPR) effect, active targeting via surface-conjugated ligands, and magnetic field-guided targeting [100].

Passive targeting takes advantage of the characteristic pathophysiology of tumor tissues, including their leaky vasculature and impaired lymphatic drainage [138]. The EPR effect allows nanoparticles within a specific size range (typically 10–200 nm) to preferentially accumulate in tumor tissues. DFMNPs, with their controllable size through dendrimer generation and magnetic core dimensions, can be optimized to maximize this passive targeting effect [129]. However, passive targeting alone may not provide sufficient selectivity for effective therapy.

Active targeting strategies enhance the specificity of drug delivery by exploiting the overexpression of certain receptors on cancer cells. Various targeting ligands can be conjugated to the dendrimer surface, including folic acid, peptides, antibodies, and aptamers. For example, folic acid conjugation has been widely studied due to the overexpression of folate receptors in many cancer types [128]. The multivalent nature of dendrimers allows for the attachment of multiple targeting ligands, potentially increasing binding avidity through the cluster effect.

**Figure 26:**
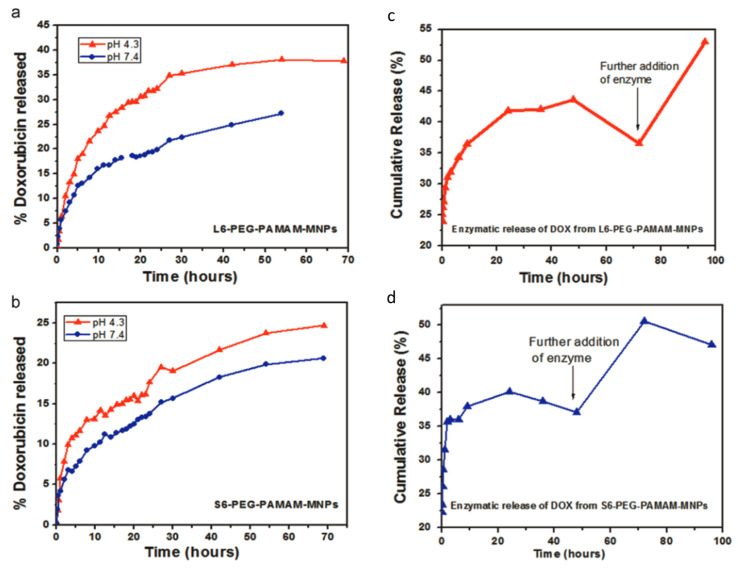
pH-dependent release of DOX from (**a**) L6-PEG-PAMAM-MNPs, (**b**) S6PEG-PAMAM-MNPs, Cathepsin B-mediated release of DOX from L6-PEG-PAMAM-MNPs (**c**), and S6-PEG-PAMAM-MNPs (**d**). Reprinted from [125], with permission from Elsevier.

The incorporation of magnetic nanoparticles enables an additional targeting mechanism through external magnetic field guidance. When exposed to an external magnetic field gradient, these systems can be concentrated at specific locations, enhancing local drug concentrations [16]. This magnetic targeting has been demonstrated to significantly improve the therapeutic efficacy of drug-loaded DFMNPs compared to non-magnetic systems (Figure 26) [125]. The combination of magnetic targeting with active targeting ligands has shown synergistic effects in several studies [129].

Recent advances have focused on developing smart targeting systems that respond to tumor-specific stimuli. For instance, pH-sensitive linkages between targeting ligands and dendrimers can enhance drug release specifically in the acidic tumor environment [132]. Some systems incorporate enzyme-responsive elements that become activated in the presence of tumor-associated enzymes, providing an additional layer of targeting specificity.

The choice of targeting strategy significantly impacts the biological performance of DFMNPs. While active targeting can enhance cellular uptake and specificity, it may also affect the pharmacokinetics and biodistribution of the nanoparticles. Similarly, magnetic targeting must be carefully optimized to balance targeting efficiency with practical clinical implementation [139]. Understanding these various targeting mechanisms and their interplay is crucial for designing effective drug delivery systems.

The controlled release of therapeutic agents from dendrimer-functionalized magnetic nanoparticles (DFMNPs) can be achieved through various stimuli-responsive mechanisms. Understanding and optimizing these release mechanisms is crucial for developing effective drug delivery systems that maximize therapeutic efficacy while minimizing side effects.

pH-responsive drug release represents one of the most widely exploited mechanisms in DFMNP systems, taking advantage of the pH gradient between normal tissues (pH 7.4) and tumor microenvironments (pH 6.5–6.8) or intracellular compartments like endosomes (pH 5.0–5.5). PAMAM dendrimers, in particular, demonstrate excellent pH-responsive behavior due to their tertiary amine-rich interior [125]. At physiological pH, these internal amines remain largely unprotonated, maintaining a compact dendrimer structure that effectively encapsulates drug molecules. However, in acidic conditions, protonation of these amines leads to conformational changes through electrostatic repulsion, facilitating drug release [132].

Figure 26 effectively illustrates the pH-responsive release profiles of doxorubicin (DOX) from dendrimer-functionalized MNPs. Panels (a) and (b) highlight the significant differences in DOX release at acidic (pH 4.3) versus physiological pH (pH 7.4), underscoring the potential of dendrimers to exploit tumor microenvironment acidity for targeted drug delivery. Additionally, panels (c) and (d) demonstrate enzymatic release triggered by Cathepsin B, showing a cumulative increase in DOX release with further enzyme addition. These findings emphasize the dual-trigger mechanism—pH and enzyme sensitivity—that enhances the efficacy of drug release systems, particularly for intracellular delivery within acidic compartments like endosomes. The data not only validates the pH-responsive behavior of PAMAM dendrimers but also highlights their adaptability for multi-modal, stimuli-responsive drug delivery systems. The pH-dependent release can be further enhanced through the incorporation of acid-labile linkages between the drug and dendrimer structure. Common approaches include the use of hydrazone, acetal, or imine bonds, which remain stable at physiological pH but undergo rapid hydrolysis in acidic environments [133]. For example, doxorubicin conjugated to DFMNPs via hydrazone bonds shows minimal release at pH 7.4 but rapid release at pH 5.5, corresponding to endosomal conditions [129].

The magnetic core of DFMNPs enables temperature-triggered release through the application of alternating magnetic fields (AMF). Under AMF, magnetic nanoparticles generate localized heat through Néel and Brownian relaxation mechanisms [140]. This localized hyperthermia can trigger drug release through various mechanisms, such as temperature-induced conformational changes in the dendrimer structure, disruption of temperature-sensitive bonds, and enhanced diffusion rates at elevated temperatures.

The combination of magnetic hyperthermia with pH-responsive release often shows synergistic effects. Elevated temperatures can accelerate the hydrolysis of pH-sensitive bonds and enhance the conformational changes of dendrimers, leading to more efficient drug release [139].

**Figure 27:**
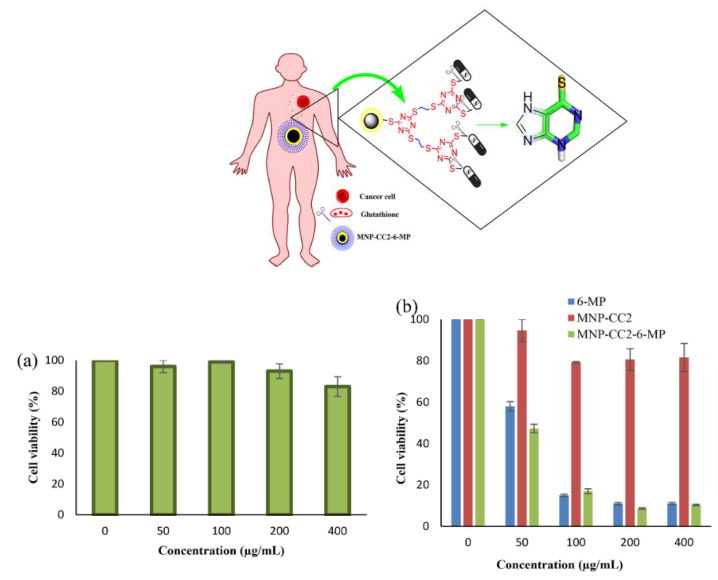
The structure of release of 6-MP from sulfur decorated dendrimer and (**a**) The effect of different concentrations of MNP@CC2 on viability L929 normal cell for 24 h and (**b**) effect of different concentrations of MNP@CC2, MNP@CC2-6-MP, and free 6-MP on the viability of MCF7 cancer cell for 72 h. Reprinted from [141], with permission from Elsevier.

Enzymatic degradation provides another mechanism for controlled release, particularly relevant for systems utilizing biodegradable linkages. Many tumor environments show elevated levels of specific enzymes, which can be exploited for targeted release. For example, cathepsin B, overexpressed in many cancer cells, can cleave specific peptide sequences, enabling selective drug release at tumor sites (Figure 27) [125,141]. The design of enzyme-responsive DFMNPs typically involves incorporating enzyme-cleavable spacers between the drug and dendrimer structure.

The biological performance of dendrimer-functionalized magnetic nanoparticles (DFMNPs) is crucial for their successful application in drug delivery. Understanding their interactions with biological systems, from cellular uptake to systemic distribution, is essential for optimizing their therapeutic efficacy and safety.

DFMNPs can enter cells through multiple endocytic pathways, with the predominant mechanism depending on particle size, surface charge, and functionalization. For PAMAM-based systems, clathrin-mediated endocytosis is often the primary route of entry, particularly for particles in the 50–200 nm size range [124]. Surface charge plays a crucial role, with positively charged dendrimers showing enhanced cellular uptake due to interactions with the negatively charged cell membrane. However, this can also lead to non-specific uptake and potential toxicity.

The presence of targeting ligands can direct DFMNPs toward specific receptor-mediated endocytic pathways. For example, folate-conjugated systems are internalized via folate receptor-mediated endocytosis, providing enhanced specificity for cancer cells overexpressing these receptors [128]. Magnetic field application can further enhance cellular uptake through magnetofection mechanisms, where the external field concentrates particles at the cell surface and promotes internalization [125].

Following internalization, DFMNPs typically follow the endolysosomal pathway, progressing from early endosomes to late endosomes and finally lysosomes. This trafficking pattern is particularly advantageous for pH-responsive drug delivery systems, as the progressively acidifying environment triggers drug release [133]. Studies using fluorescently labeled dendrimers have revealed that while the dendritic portion may escape the endosomes, the magnetic core often remains within vesicular compartments.

The fate of DFMNPs inside cells depends significantly on their surface properties. Some systems demonstrate the ability to escape endosomes through the “proton sponge effect”, particularly with PAMAM dendrimers, which can buffer the endosomal pH and cause osmotic swelling and rupture of the endosomal membrane [129].

The cytotoxicity of DFMNPs must be carefully evaluated to ensure their safety for therapeutic applications. Studies typically assess both the empty carrier system and drug-loaded formulations. Generation-dependent toxicity has been observed with PAMAM dendrimers, where higher generations tend to show increased cytotoxicity due to their greater number of surface groups [132]. Surface modification strategies, such as PEGylation or acetylation of surface amines, can significantly reduce this inherent toxicity.

Drug-loaded systems often demonstrate enhanced cytotoxicity against cancer cells compared to free drugs, attributed to improved cellular uptake and controlled release properties. For instance, doxorubicin-loaded DFMNPs have shown significantly lower IC50 values in various cancer cell lines compared to free doxorubicin, particularly when combined with magnetic targeting [125].

The hemocompatibility of DFMNPs is critical for their intravenous administration. The key parameters evaluated include hemolysis assays to assess red blood cell damage, platelet activation and aggregation studies.

Studies have shown that surface modification of dendrimers can significantly improve blood compatibility. Neutral or negatively charged surface groups generally show better hemocompatibility compared to positive charges [139]. The incorporation of biocompatible coatings like PEG can further reduce interactions with blood components.

The biodistribution of DFMNPs is influenced by various physicochemical properties and can be tracked using multiple imaging modalities, taking advantage of their magnetic core. Generally, particles in the 10–100 nm size range show prolonged circulation times and enhanced tumor accumulation through the EPR effect [142]. However, a significant portion often accumulates in the liver and spleen due to RES uptake.

Magnetic targeting can significantly alter the biodistribution pattern, enhancing accumulation at target sites. Studies using magnetic resonance imaging have demonstrated an increased tumor uptake of DFMNPs under applied magnetic fields [16]. The clearance pathway depends largely on particle size and surface properties, with smaller particles (<5–6 nm) showing renal clearance while larger ones undergo hepatobiliary elimination.

The optimization of targeting strategies for DFMNPs requires careful consideration of multiple interrelated factors that affect their biological performance. The addition of targeting ligands, while enhancing specificity, can significantly alter the nanoparticle surface properties and consequently their biological interactions. For instance, the conjugation of targeting molecules may change the surface charge or hydrophobicity of DFMNPs, affecting their protein corona formation and circulation time [143]. Surface modification with targeting ligands can also influence the rate of reticuloendothelial system (RES) recognition and clearance, potentially reducing the therapeutic window [129].

Magnetic targeting presents its own set of optimization challenges. While external magnetic fields can effectively concentrate DFMNPs at target sites, the strength and duration of magnetic field application must be carefully controlled. Excessive magnetic field strengths may lead to aggregation of nanoparticles, potentially causing embolization in blood vessels [16]. Additionally, the effectiveness of magnetic targeting decreases with tissue depth and blood flow rate, making it challenging to target deep-seated tumors [140].

The combination of multiple targeting strategies requires careful consideration of their potential interactions. For example, the presence of targeting ligands may affect the magnetic responsiveness of DFMNPs by altering their hydrodynamic size and surface properties. Conversely, magnetic field application might influence the binding kinetics of targeting ligands to their receptors [125]. Understanding these complex interactions is crucial for optimizing targeting efficiency.

Practical clinical implementation introduces additional considerations. The magnetic field strength and gradient required for effective targeting must be achievable with clinically approved devices while maintaining patient safety and comfort. The positioning and duration of magnetic field application need to be optimized for different tumor locations and patient anatomies [142]. Furthermore, the manufacturing complexity and cost implications of multiple targeting strategies must be balanced against their therapeutic benefits.

Recent studies have focused on developing “smart” targeting systems that can adapt to biological conditions. For instance, some designs incorporate stimuli-responsive linkers that expose targeting ligands only in the tumor microenvironment, potentially improving the targeting specificity while minimizing off-target effects during circulation [132]. These advanced designs show promise but require extensive validation before clinical translation.

## 6. Conclusions and Future Outlook

The development of nanocarrier drug delivery systems for cancer treatment has been a promising topic for biomedical researchers and oncologists, and the properties of MNPs have made them an alluring modification to nanocarriers by adding functionalities that can help overcome certain limitations. This review discussed different form factors of nanocarriers—which were hydrogels, lipidic vesicles ranging from liposomes to exosomes, and dendrimers—and how these nanocarriers function in drug delivery, how they are enhanced with MNP incorporation, and what specific cancer applications the MNP-coupled nanocarriers are being researched for. For all these nanocarriers, targeted drug delivery can be a significant advantage to increase drug accumulation in tumors while minimizing the cytotoxicity of the drug to healthy cells. The magnetic properties of MNPs have demonstrated the ability to enhance the targeting aspects of nanocarriers to tumors through magnetic guidance. MNPs also possess hyperthermia capabilities when exposed to alternating magnetic fields, which can augment the nanocarriers by enabling controlled release by magnetically inducing enough heat to release the drugs from the nanocarrier molecule. In addition, nanocarriers can possess double functionality with MNPs by delivering MNPs to cancer sites which can be thermally ablated by magnetic hyperthermia. Together with the stability and deep-tumor penetration capabilities of hydrogels, the high biocompatibility and prolonged circulation offered by lipidic vesicles, and versatile properties and capabilities of dendrimers; MNP-nanocarrier systems have displayed promising results for numerous anti-cancer applications ranging from breast cancer to liver cancer and many more.

Despite the advancements offered by MNP integration, there remain challenges and limitations that need to be addressed for nanocarriers before they can be clinically adopted. By far the biggest limitation is the need for more in vivo and clinical trials to observe the potential long-term effects and cytotoxicity of exposing patients to these drug delivery systems. While magnetically enhanced targeting shows potential, the technique can be limited by the tumor location in the body and how deep-seated they are. In addition, researchers still need to improve their understanding of the accumulation of MNPs in non-target tissues and how these MNPs are eventually cleared from the body after treatment. Each nanocarrier still possesses certain limitations even with MNPs incorporation, such as the dispersion of hydrophobic drugs from hydrogels, the stability and immune system detection of certain lipidic vesicles, and the effects of surface modification and reduced circulation time of dendrimers. Lastly, the scalability and cost of these MNP nanocarriers need to be considered against their potential efficacy. If these augmented nanocarriers prove to be too expensive to produce in abundant quantity, this will certainly diminish the future outlook for this technology.

Overall, MNPs offer great potential to augment current drug delivery systems and nanocarriers to improve their performance in a variety of anti-cancer applications. Each MNP-augmented nanocarrier that has been discussed demonstrates a unique specialty or advantage for treating certain tumors or a range of tumors and cancers. These advantages include magnetic guidance, deep tumor penetration, and magnetic hyperthermia to increase effectiveness against resistant tumors and increase specificity of chemotherapeutic agents. Currently, most of these MNP-augmented drug delivery systems remain as pre-clinical modalities that require substantially more research into in vivo efficacy, safety, and scalability before adoption in the clinical setting. With more time and research, MNP-augmented drug delivery systems could surely aid in the ongoing battle against cancer by providing improved specificity and effectiveness of chemotherapeutic drugs in treatment.
